# Epidemic Changes in the Local Affections of Fever

**Published:** 1855-08

**Authors:** 


*Epidemic Changes in the Local Affections of Fever.* By Dr. Stokes.

In their seat, if not in their nature, these affections are observed to vary in different countries. On the continent—at least in France, and in a large proportion of Germany—the frequency, and, probably, the preponderance of the secondary disease of the intestines, is a matter that must be admitted. So remarkable, indeed, is the predominance of the tumefaction and ulceration of the mucous glands of the intestines in France, that Andral, in the first edition of the *Clinique Médicale,* described fevers under the general head of diseases of the digestive system; and yet Andral was no blind follower of Broussais. In Ireland, however, we do not find this remarkable preponderance of the secondary diseases of the digestive system; but, when I state this to you, I wish you to understand and adopt this principle, that all statements as to the anatomical characters of fever, as it prevails here or elsewhere, are to be accepted only so far as they apply to the prevailing epidemic. And, although it is true that on comparing our typhus with the French typhoid fever, this difference becomes apparent, that the existence of follicular disease of the intestine is almost the rule, and its absence the exception in the latter affection, while, in the Irish typhus, this condition of the intestine is rare; you must, however, bear in mind, that in Ireland, and in our own time, we have had a great epidemic of what was certainly typhus fever, in which the condition of the intestine accurately represented that which is found to prevail on the continent.—*Ranking's Abstract.*

